# A deep learning-based framework for standardized analysis of trabecular bone compartments from micro-CT imaging data in the mouse tibia

**DOI:** 10.1038/s41598-025-19698-w

**Published:** 2025-10-14

**Authors:** Amine Lagzouli, Lucinda Evans, Mark Hopkinson, Aikta Sharma, Natalia M. Castoldi, Davide Fontanarosa, Maria Antico, David M. L. Cooper, Alice Othmani, Vittorio Sansalone, Phil Salmon, Andrew A. Pitsillides, Peter Pivonka

**Affiliations:** 1 School of Mechanical, Medical, and Process Engineering, Queensland University of Technology, Gardens Point Campus, 2 George St, 4000 Brisbane, QLD Australia; 2 Univ Paris Est Creteil, Univ Gustave Eiffel, CNRS, UMR 8208, MSME, 94010 Créteil, France; 3 Department of Comparative Biomedical Sciences, Royal Veterinary College, NT1 0TU London, UK; 4 Department of Mechanical Engineering, University College London, London, UK; 5 Department of Anatomy, Physiology, and Pharmacology, University of Saskatchewan, Saskatoon, Saskatchewan Canada; 6 Université Paris-Est Créteil (UPEC), LISSI, Vitry sur Seine, 94400 Créteil, France; 7 School of Clinical Sciences, Queensland University of Technology, Gardens Point Campus, 2 George St, 4000 Brisbane, QLD Australia; 8 Centre for Biomedical Technologies (CBT), Queensland University of Technology, Brisbane, 4000 QLD, Australia; 9 CSIRO Health and Biosecurity, The Australian eHealth Research Centre, 4029 Herston, QLD Australia; 10 Bruker Belgium (microCT), Preclinical Imaging, Kontich, Belgium

**Keywords:** Trabecular bone, Deep learning, Micro-computed tomography (micro-CT), Mouse tibia, Preclinical research, Preclinical research, Machine learning, Computer science, Bone, Imaging

## Abstract

Understanding bone remodeling and disease progression is crucial in preclinical skeletal research, particularly for assessing pharmacological and mechanical interventions in the long bones of murine models. High-resolution micro-computed tomography (micro-CT) imaging enables detailed trabecular bone analysis; however, inconsistent and non-standardized definitions of the volumes of interest (VOIs) across the different trabecular compartments compromise reproducibility and may lead to misleading statistical interpretations. In this study, we introduce a deep learning framework for automated trabecular bone analysis from micro-CT scans (5 µm voxel size) of the epiphyseal-metaphyseal region in the mouse tibia. The epiphyseal-metaphyseal region is classified into four anatomical compartments, epiphyseal bone, growth plate, primary spongiosa, and secondary spongiosa, using a 2D slice-wise classification model combined with a regional probability distribution method to detect the transitional landmarks between these compartments and enable standardized VOI extraction. To validate our method, we trained and tested the model on three micro-CT datasets comprising a total of 40 bone scans, each annotated by three experts to assess inter- and intra-operator variability, and further assessed its generalizability using an additional external dataset. These datasets encompassed diverse experimental conditions, including pharmacological treatments, mechanical loading, and age-related reduced bone density. Our classification model achieved excellent performances (mean F1-score of 0.96 for the epiphyseal bone, 0.95 for the growth plate, 0.92 for the primary spongiosa, and 0.99 for the secondary spongiosa across all datasets; statistical equivalence within 0.05 mm, $p \le 0.05$) and demonstrated strong generalizability on the external dataset (mean F1-score of 0.99 for the epiphyseal bone, 0.97 for the growth plate, 0.92 for the primary spongiosa, and 1.0 for the secondary spongiosa; statistical equivalence within 0.05 mm, $p \le 0.05$). Following the extraction of the different trabecular compartments, we segmented the trabecular bone within the epiphyseal bone, primary spongiosa, and secondary spongiosa using a deep learning-based segmentation model. We performed a comprehensive morphological and statistical analysis of all trabecular compartments in the mouse tibia, facilitating consistent comparisons across experimental groups and enabling direct comparisons within and between trabecular compartments. This automated method provides a consistent and robust tool for analyzing micro-CT scans of the trabecular bone in the mouse tibia, facilitating advancements in preclinical skeletal research.

## Introduction

The precise and efficient analysis of trabecular bone compartments in murine models is critical for advancing skeletal research, particularly in understanding disease progression and evaluating the efficacy of therapeutic interventions, such as pharmaceuticals or exercise regimens^1,2^. Preclinical studies on degenerative skeletal conditions such as osteoporosis and osteoarthritis often involve detailed characterization of trabecular bone near key joints, such as the hip and knee, and are typically studied in long bones, namely the tibia and femur, in preclinical rodent models^3–5^. These analyses rely on morphometric assessments to characterize changes across distinct anatomical volumes of interest (VOIs). Specifically, the epiphyseal-metaphyseal region of long bones can be divided into four compartments: (i) epiphyseal bone, (ii) growth plate, (iii) primary spongiosa, and (iv) secondary spongiosa. Osteoarthritis studies primarily focus on epiphyseal bone changes^6–8^, whereas studies focusing on osteoporosis examine alterations in the metaphyseal bone (secondary spongiosa alone or mixed primary-secondary spongiosa)^2,9–11^.

The secondary spongiosa is traditionally the region analyzed in long bones, as it exists on a larger spatio-temporal scale and persists for longer durations than the newly formed primary spongiosa. To isolate the secondary spongiosa, an offset distance from the growth plate is commonly used to exclude the primary spongiosa^2,11^. For mice, the offset is usually around 0.2–0.3 mm^12,13^, while for rats it is approximately 0.4–1.5 mm^14,15^. The concept of an imposed offset for analyzing trabecular bone in the secondary spongiosa originally emerged from 2D histomorphometric principles^16–21^. This has since been adapted for 3D micro-computed tomography (micro-CT) imaging^2,11–13,22–28^. Recently, Salmon et al.^2^ have concluded that analyzing the primary and secondary spongiosa combined adds a valuable dimension to histomorphometric analysis, further emphasizing the need for precise localization of these compartments. Nevertheless, the location of the four compartments in the epiphyseal-metaphyseal region of long bones varies across studies, as it depends on factors such as age, size, sex, pharmacological treatments, and mechanical loading (ML) among others^2,11^.

Identifying these compartments from micro-CT imaging is inherently challenging, typically requiring manual extraction of VOIs for each compartment and subsequent segmentation of trabecular bone within these VOIs, a time-consuming process susceptible to user-dependent variability. Furthermore, the absence of standardized protocols for defining these compartments and their corresponding VOIs compromises reliable statistical analyses. Currently, there is no consensus or standardized approach for selecting the VOIs in the context of trabecular bone analysis in preclinical research. Different research groups have examined distinct VOIs in the tibia or the femur, reflecting methodological variations and specific experimental objectives, which compromises the comparability of morphological parameters across different studies. In a series of experiments, Sugiyama et al. investigated ML and pharmacological interventions within different VOIs of the tibial spongiosa. Sugiyama et al. (2008)^22^ focused on the influence of intermittent parathyroid hormone (PTH) and ML on the primary and secondary spongiosa, analyzing regions extending 0.01–0.25 mm and 0.25–1.25 mm distal to the growth plate, respectively. Subsequently, Sugiyama et al. (2011)^12^ examined the secondary spongiosa extending 0.25–0.75 mm distal to the growth plate, to evaluate risedronate effects on loading-induced increases in trabecular bone mass. Finally, Sugiyama et al. (2012)^23^ explored ML under varying intensities following sciatic neurectomy (SN), analyzing only the secondary spongiosa extending 0.25–0.75 mm distal to the growth plate. Other studies have analyzed trabecular bone in the mouse tibia across a spectrum of distinct VOIs. For example, Meakin et al.^24^ analyzed the effects of SN and ageing in the secondary spongiosa in a VOI of 0.25–0.75 mm distal to the proximal physis. Roberts et al.^13^ analyzed the effects of PTH and ML on trabecular bone in ovariectomized mice, using a 1 mm VOI starting from the most distal slice that included the growth plate, as adapted from van’t Hof et al^10^. Willie et al.^25^ demonstrated that adult mice exhibited a reduced and delayed trabecular bone response to ML compared to younger mice, analyzing the secondary spongiosa in a proximal metaphyseal VOI extending 5% of tibial length, starting 0.25 mm below the growth plate. Monzem et al.^26^ investigated the region-specific modular responses of different bone compartments to PTH, focusing on trabecular bone within the proximal 6–16% of the total tibial length. There is also no consensus regarding the height of the selected volume of interest in the secondary spongiosa. Some studies define it as a fixed distance from the growth plate, such as 0.5 mm^12,22,24^ or 1.0 mm^10,13,22^. Other approaches define the region of interest based on a percentage of the bone length, typically ranging from 5–10%^26–28^.

These variations highlight a notable inconsistency in critical parameters across studies, including the precise location of the growth plate, the offset distances to the secondary spongiosa and epiphyseal bone, and the height of the VOI used for trabecular bone analysis in the epiphyseal-metaphyseal region of the mouse tibia. This lack of standardization limits the reproducibility of individual studies and complicates cross-study comparisons, as each investigation examines a different VOI. A standardized approach to defining these parameters is essential to ensure robust and comparable results in preclinical skeletal research. Recently, deep learning approaches have emerged as transformative tools for bone image analysis in multiple applications in preclinical translational research^29–35^. Neetson et al.^32^ proposed a fully automated algorithm for the segmentation of cortical and trabecular compartments in HR-pQCT scans of the human radius and tibia. Lagzouli et al.^33^ introduced a novel hybrid neural network architecture, the Dual-Branch Attention-based Hybrid Network (DBAHNet), for high-resolution micro-CT bone scan segmentation. Trained on a limited control dataset, DBAHNet achieved excellent performance on a large and diverse dataset from different research studies^34^. More recently, Burlutskiy et al.^35^ adopted a deep learning approach to identify the growth plate in micro-CT scans of rodents, which is essential for fully automatic segmentation of trabecular bone for preclinical drug development studies.

In this paper, we introduce a deep learning-based framework for robust and consistent VOI extraction, segmentation, and analysis of the different trabecular bone compartments from micro-CT scans (5 µm) of the epiphyseal-metaphyseal region in mouse tibiae. We trained a deep learning-based classification model to classify cross-sectional 2D slices from micro-CT images of mouse tibiae into four key compartments: epiphyseal bone, growth plate, primary spongiosa, and secondary spongiosa. Additionally, we proposed a regional probability distribution approach to detect the three transitional landmarks between these compartments: $Z_{eg}$ represents the transitional interface between the epiphyseal bone and the growth plate, $Z_{gp}$ represents the transitional interface between the growth plate and the primary spongiosa, and $Z_{ps}$ represents the transitional interface between the primary and secondary spongiosa. This enables a consistent extraction of standardized VOIs of the trabecular bone compartments across different experimental groups. This method is widely applicable, supporting studies on epiphyseal bone for cartilage and osteoarthritis research, as well as analyses of the secondary spongiosa alone or combined with the primary spongiosa in the metaphysis for osteoporosis and bone remodeling research. We validated the classification model using three micro-CT mouse tibia datasets^22–24^, totaling 40 bone scans. These datasets encompassed pharmacological interventions, mechanical loading variations, and aged mice with minimal trabecular bone. Manual annotations from three independent human raters were used as ground truth, and the model achieved a mean F1-score of 0.96 for the epiphyseal bone, 0.95 for the growth plate, 0.92 for the primary spongiosa, and 0.99 for the secondary spongiosa across all datasets. The axial positions of the transitional landmarks were statistically equivalent to those identified by at least two manual annotators, within a 0.05 mm margin (p $\le$ 0.05). To assess generalizability, we evaluated the model on an external dataset^12^ comprising two groups: risedronate treatment alone and risedronate combined with ML. The model achieved a mean F1-score of 0.99 for the epiphyseal bone, 0.97 for the growth plate, 0.92 for the primary spongiosa, and 1.0 for the secondary spongiosa, and the axial positions of the predicted transitional landmarks were statistically equivalent to manual annotation within a 0.05 mm margin (p $\le$ 0.05). Subsequently, deep learning-based segmentation was performed within standardized VOIs for each group, segmenting the epiphyseal trabecular bone, primary spongiosa, and secondary spongiosa. These segmentations were then used to conduct a comprehensive cross-sectional and 3D morphological and statistical analysis of the trabecular bone compartments, facilitating consistent comparisons across experimental groups and enabling direct comparisons within and between trabecular compartments. We further investigated the effects of inconsistent VOI definitions, particularly in the secondary spongiosa, by comparing our anatomically defined VOIs with the conventional fixed-offset approach downstream of the growth plate. Our results highlight the limitations of this traditional method, demonstrating its potential to undermine consistency and to lead to misleading statistical interpretations in trabecular bone analysis. The proposed method enables automated, robust, and consistent analysis of trabecular compartments in the epiphyseal-metaphyseal region of the tibia in murine models, accelerating preclinical skeletal research and the assessment of drug treatment effectiveness.

## Results

### Performance evaluation of the proposed method

We performed 5-fold cross-validation during the evaluation of the classification model’s performance against the manual annotation using test sets from three datasets: Dataset 1^22^, Dataset 2^23^, and Dataset 3^24^, as reported in Table 1. The model demonstrated strong overall performance, accurately classifying cross-sectional 2D slices of micro-CT scans from the epiphyseal-metaphyseal region of the mouse tibia. Across all datasets, the model achieved a mean F1-score of 0.96 for the epiphyseal bone, 0.95 for the growth plate, 0.92 for the primary spongiosa, and 0.99 for the secondary spongiosa. In Dataset 1^22^, the model performed robustly across all classes, with F1-scores of 0.96 for the epiphyseal bone, 0.95 for the growth plate, 0.93 for the primary spongiosa, and 0.99 for the secondary spongiosa. Performance remained consistently high across all PTH treatment groups (PTH0 to PTH80). In Dataset 2^23^, the model maintained high classification performance, achieving F1-scores of 0.96 for the epiphyseal bone, 0.95 for the growth plate, 0.91 for the primary spongiosa, and 0.99 for the secondary spongiosa. Model predictions remained high across ML conditions with varying peak dynamic load magnitudes under SN (0N, 6 N, and 12 N). In Dataset 3^24^, which included aged mice with significantly reduced bone volume fraction, the model achieved F1-scores of 0.96 for the epiphyseal bone, 0.95 for the growth plate, 0.90 for the primary spongiosa, and 0.99 for the secondary spongiosa. Notably, the model consistently achieved the highest performance in the epiphyseal bone and secondary spongiosa compartments across all datasets, which are key compartments for trabecular bone analysis. The secondary spongiosa reached a mean F1-score of 0.99, while the epiphyseal bone maintained a mean F1-score of 0.96, highlighting the reliability of the classification. Although classification performance for the primary spongiosa was modestly lower in Dataset 2 and Dataset 3 (F1-scores ranging from 0.90 to 0.91), this is likely due to the limited presence of the primary spongiosa in those datasets. In such cases, the focus on secondary spongiosa alone remains appropriate for metaphyseal bone analysis, consistent with the standard study protocols^9,10^. This was not the case in groups with abundant primary spongiosa, where classification performance remained strong across different PTH dose groups in Dataset 1^22^, underscoring its robustness across diverse trabecular architectures.

**Fig. 1:**
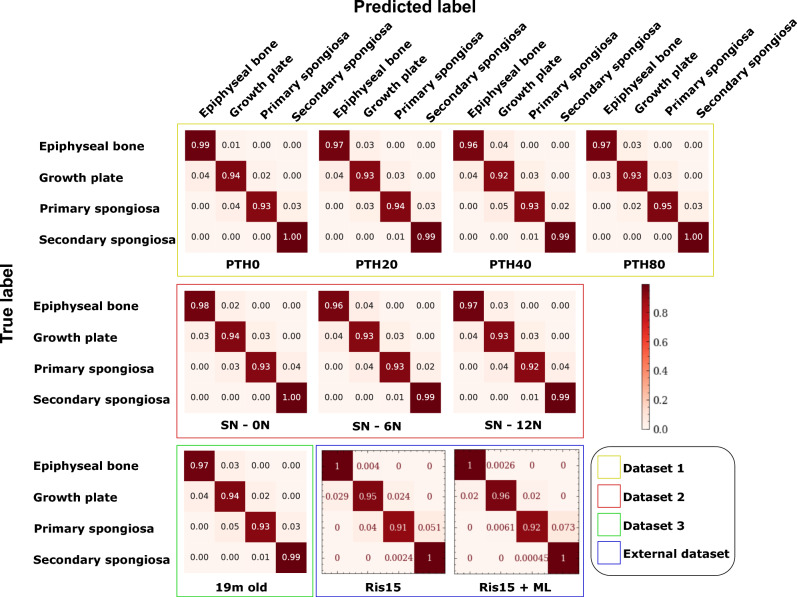
Performance evaluation of the deep-learning classification model for classifying 2D micro-CT slices from the epiphyseal-metaphyseal region of the mouse tibia to four compartments: epiphyseal bone, growth plate, primary spongiosa, and secondary spongiosa, using confusion matrices. The confusion matrices show mean results across five-fold cross-validation. Evaluation was conducted on all experimental groups in Dataset 1^22^, Dataset 2^23^, Dataset 3^24^, and the external dataset^12^. Group abbreviations are as follows: PTH0, PTH20, PTH40, and PTH80 indicate parathyroid hormone doses of 0 to 80 µg/kg/day; SN denotes sciatic neurectomy; Ris15 indicates risedronate at 15 µg/kg/day; and ML denotes mechanical loading.

To further analyze the model’s performance, confusion matrices were examined across different datasets and groups (Fig. 1). The model exhibited classification accuracies approaching 100% for epiphyseal bone and secondary spongiosa. Across all groups and datasets, the secondary spongiosa consistently achieved very high accuracy, with only a few slices misclassified as the adjacent anatomical region (i.e., primary spongiosa), demonstrating the effectiveness of the proposed method. Similarly, the epiphyseal bone exhibited excellent classification accuracy, with only minor misclassifications occurring in the adjacent growth plate region. While classification accuracy remained high for the growth plate and primary spongiosa, occasional misclassifications occurred at the interfaces between these regions, but the overall results were excellent as illustrated in Table 1. For instance, in the PTH0 group (i.e. vehicle treated), the model correctly classified 94% of the growth plate instances. The remaining false positives were misclassified as 4% epiphyseal bone and 2% primary spongiosa. Such misclassifications is expected due to the inherent limitations of cross-sectional analysis in the proximal tibia. As certain slices contain a mixture of adjacent regions, the model encounters uncertainty when assigning class labels, leading to minor errors at anatomical boundaries as discussed above. Transitional zones naturally exist between the anatomically defined compartments, resulting in overlapping features across adjacent regions^2,11^. This structural continuity introduces minor ambiguity not only in automated classification but also in the manual approach.

To demonstrate the model’s ability to generalize to external data under different experimental setups, we retrained the model on all three datasets to obtain the final model, and tested it on the external dataset^12^. This dataset included two distinct mice groups: one treated with risedronate (15 µg/kg/day) alone, and the other with risedronate combined with ML. As shown in Table 1, the model maintained strong classification performance across all compartments, achieving a mean F1-score of 0.99 for the epiphyseal bone, 0.97 for the growth plate, 0.92 for the primary spongiosa, and 1.0 for the secondary spongiosa. The confusion matrices in Fig. 1 illustrate near-perfect classification in both groups, particularly for the epiphyseal bone and secondary spongiosa. Despite variations in bone morphology and experimental conditions, the model extracted anatomical landmarks consistently, demonstrating its robustness in trabecular bone analysis. These results confirm that the model effectively generalized to unseen experimental conditions while maintaining high classification accuracy and reliability across all the compartments in the epiphyseal-metaphyseal region of the proximal tibia in the mouse.

**Table 1:** Performance evaluation of the deep-learning classification model for classifying 2D micro-CT slices from the epiphyseal-metaphyseal region of the mouse tibia to four compartments: epiphyseal bone, growth plate, primary spongiosa, and secondary spongiosa. Metrics reported are the mean values across five-fold cross-validation, including Precision, Recall, and F1-score. Results are presented for all experimental groups in Dataset 1^22^, Dataset 2^23^, Dataset 3^24^, and the external dataset^12^. Group abbreviations are as follows: PTH0, PTH20, PTH40, and PTH80 indicate parathyroid hormone doses of 0 to 80 µg/kg/day; SN denotes sciatic neurectomy; Ris15 indicates risedronate at 15 µg/kg/day; and ML denotes mechanical loading.

Dataset	Treatment	Metric	Epiphyseal bone	Growth plate	Primary spongiosa	Secondary spongiosa
Dataset 1^22^	PTH0	Precision	0.941	0.974	0.912	0.994
		Recall	0.986	0.943	0.917	0.995
		F1-score	0.963	0.958	0.914	0.995
	PTH20	Precision	0.939	0.957	0.933	0.994
		Recall	0.969	0.932	0.938	0.994
		F1-score	0.954	0.944	0.935	0.994
	PTH40	Precision	0.935	0.937	0.901	0.996
		Recall	0.957	0.922	0.926	0.990
		F1-score	0.946	0.930	0.913	0.993
	PTH80	Precision	0.954	0.963	0.934	0.992
		Recall	0.968	0.935	0.946	0.996
		F1-score	0.960	0.949	0.940	0.994
Dataset 2^23^	SN - 0N	Precision	0.958	0.969	0.893	0.993
		Recall	0.976	0.939	0.919	0.995
		F1-score	0.967	0.954	0.906	0.994
	SN - 6N	Precision	0.941	0.946	0.912	0.994
		Recall	0.961	0.926	0.933	0.992
		F1-score	0.951	0.936	0.922	0.993
	SN - 12N	Precision	0.948	0.962	0.885	0.994
		Recall	0.975	0.933	0.910	0.994
		F1-score	0.961	0.947	0.897	0.994
Dataset 3^24^	19 m old	Precision	0.947	0.962	0.888	0.995
		Recall	0.975	0.939	0.914	0.993
		F1-score	0.960	0.950	0.901	0.994
External dataset^12^	Ris15	Precision	0.978	0.975	0.910	0.997
		Recall	0.996	0.947	0.910	0.998
		F1-score	0.987	0.961	0.910	0.997
	Ris15 + ML	Precision	0.982	0.994	0.950	0.995
		Recall	0.997	0.960	0.921	0.999
		F1-score	0.989	0.977	0.935	0.997

**Fig. 2:**
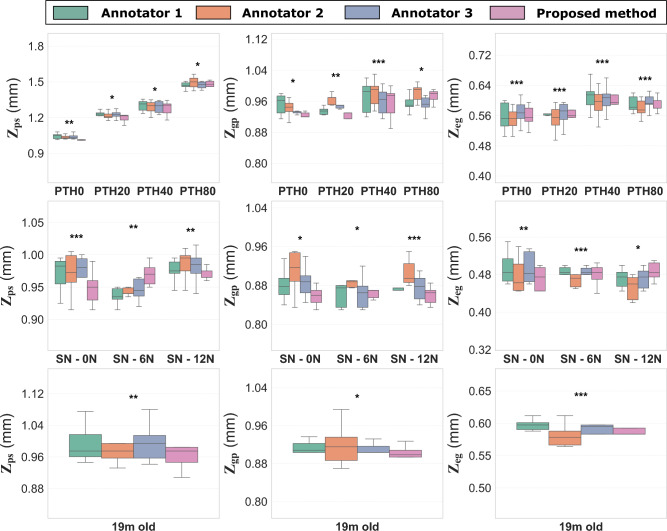
Performance evaluation of the proposed method in detecting the transitional landmarks between the different compartments in the epiphyseal-metaphyseal region of the mouse tibia for all experimental groups in Dataset 1^22^, Dataset 2^23^, Dataset 3^24^. $Z_{eg}$ represents the transitional interface between the epiphyseal bone and the growth plate. $Z_{gp}$ represents the transitional interface between the growth plate and the primary spongiosa, and $Z_{ps}$ represents the transitional interface between the primary and secondary spongiosa. Reported results represent the mean values across five-fold cross-validation. Group abbreviations are as follows: PTH0, PTH20, PTH40, and PTH80 indicate parathyroid hormone doses of 0 to 80 µg/kg/day; SN denotes sciatic neurectomy; and ML denotes mechanical loading. Statistical equivalence was assessed using Two One-Sided Tests (TOST) with an equivalence bound of ± 0.05 mm and Bonferroni correction for multiple comparisons. Asterisks indicate adjusted $p$-values for statistical equivalence between the model and at least two human annotators: *** $p \le 0.001$, ** $0.001 < p \le 0.01$, and * $0.01 < p \le 0.05$.

**Fig. 3:**
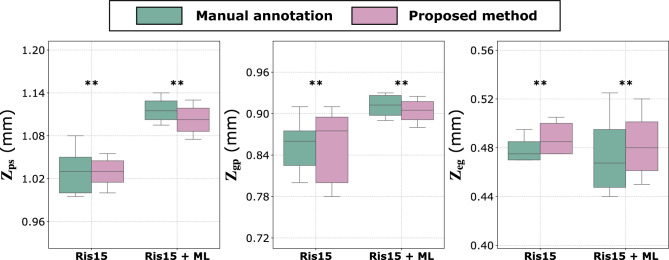
Performance evaluation of the proposed method in detecting the transitional landmarks between the different compartments in the epiphyseal-metaphyseal region of the mouse tibia in the external dataset^12^. $Z_{eg}$ represents the transitional interface between the epiphyseal bone and the growth plate. $Z_{gp}$ represents the transitional interface between the growth plate and the primary spongiosa, and $Z_{ps}$ represents the transitional interface between the primary and secondary spongiosa. Results are presented for two experimental groups: Ris15 and Ris15 + ML. Ris15 refers to a risedronate dose of 15 µg/kg/day, and Ris15 + ML refers to the same dose combined with mechanical loading (ML). Predictions are compared against manual annotations. Statistical equivalence was assessed using Two One-Sided Tests (TOST) with an equivalence bound of ± 0.05 mm. Asterisks indicate $p$-values for statistical equivalence between the model and the manual annotation: *** $p \le 0.001$, ** $0.001 < p \le 0.01$, and * $0.01 < p \le 0.05$.

**Fig. 4:**
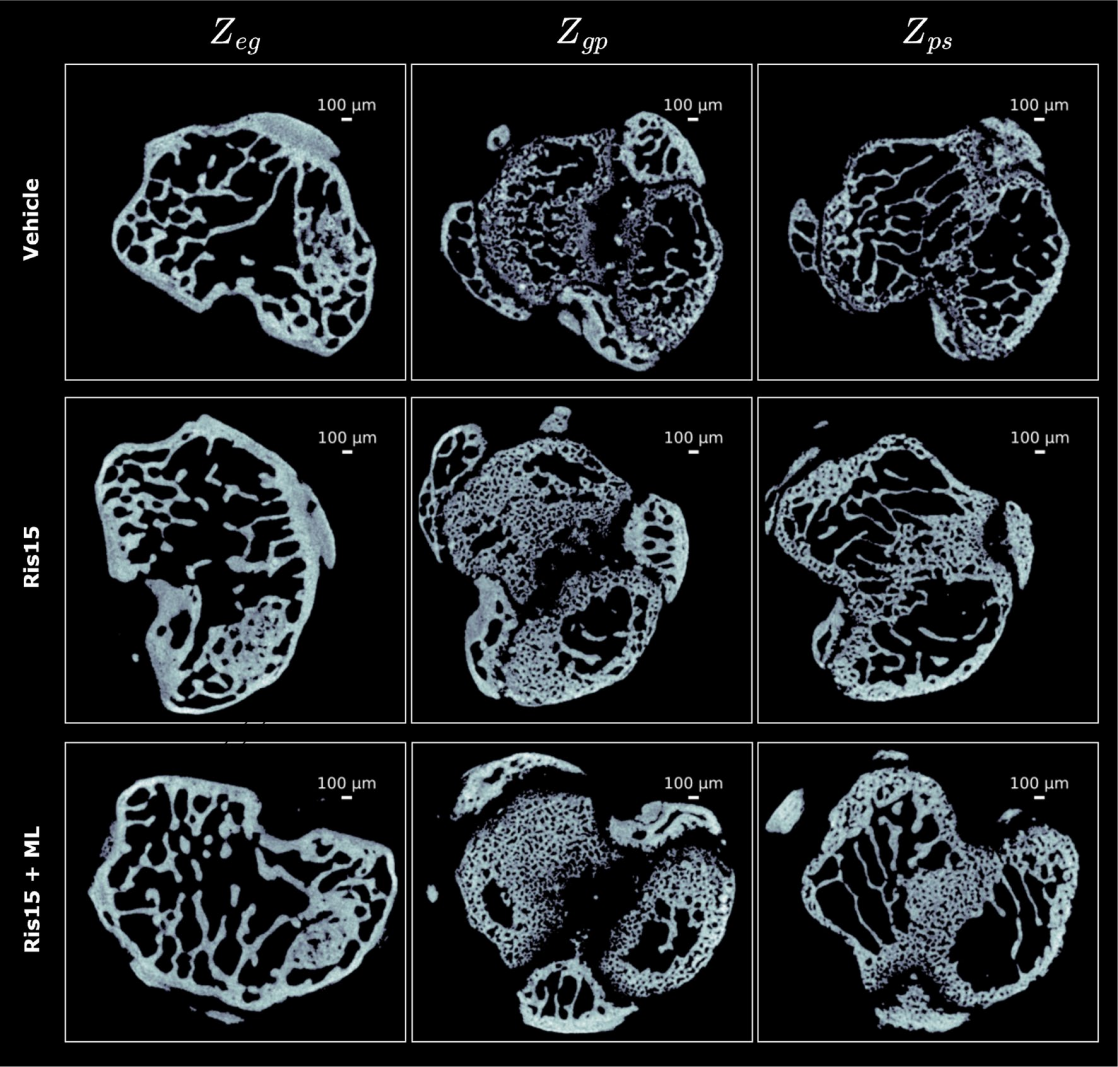
Visualisation of the micro-CT cross-sectional slices at the predicted transitional landmarks between the different compartments in the epiphyseal-metaphyseal region of the mouse tibia in the external dataset^12^. $Z_{eg}$ represents the transitional interface between the epiphyseal bone and the growth plate. $Z_{gp}$ represents the transitional interface between the growth plate and the primary spongiosa, and $Z_{ps}$ represents the transitional interface between the primary and secondary spongiosa. Results are presented for the vehicle group (top), and two experimental groups: Ris15 (middle) and Ris15 + ML (bottom). Ris15 refers to a risedronate dose of 15 µg/kg/day, and Ris15 + ML refers to the same dose combined with mechanical loading (ML).

### Transitional landmarks extraction

We further assessed the performance of the proposed method in identifying the transitional landmarks between the different compartments: the transitional interface between the epiphyseal bone and the growth plate ($Z_{eg}$), the transitional interface between the growth plate and the primary spongiosa ($Z_{gp}$), and the transitional interface between the primary and secondary spongiosa ($Z_{ps}$) along the tibia’s longitudinal axis (Z=0 denotes the proximal end of the tibia). We compared the predicted values with the manual annotations provided by three expert annotators. These annotators are researchers with expertise in bone segmentation and anatomical landmark identification (see Fig. 2). The results indicate that the model predicts these landmarks consistently across all groups and datasets, closely matching the expert annotations. The model demonstrates similar or lower variability in landmark measurements compared to the experts in nearly all groups, with an inter-operator mean intra-class correlation coefficient (ICC) of 0.98 for $Z_{eg}$ 0.94 for $Z_{gp}$, and 0.99 for $Z_{ps}$ across all groups for Dataset 1^22^. The deviation of these measurements remains stable across different groups and is, in most cases, equal to or lower than that observed among expert annotations. For each dataset and across all three landmarks, statistical equivalence within a 0.05 mm margin (p $\le$ 0.05) was established using the two one-sided t-tests (TOST). Equivalence was demonstrated both among the annotators and between the proposed model and at least two of the annotators.

The model maintains excellent performances even in cases with high drug dose, such as the PTH80-treated group, where a substantial amount of primary spongiosa was deposited downstream of the growth plate, the model’s measurement of $Z_{ps}$ (1.48 ± 0.025 mm) remained consistent with the measurements from all three annotators: Annotator 1 (1.47 ± 0.03 mm), Annotator 2 (1.50 ± 0.05 mm), and Annotator 3 (1.47 ± 0.03 mm). At the other end of the spectrum, in the 19-month-old mice from Dataset 3^24^ that have significantly reduced bone mass, the model’s $Z_{ps}$ (0.97 ± 0.05 mm) closely matched the expert annotations, all ranging between 0.97 mm and 0.99 mm ± 0.05 mm. This is also the case for the other landmarks $Z_{eg}$ and $Z_{gp}$ where the proposed approach provides a consistent and more robust method for extracting these compartments. The model achieves mean values and standard deviations that closely align with those of the expert annotators (see Fig. 2) across diverse conditions, including different doses of PTH (0, 20, 40, and 80 µg/kg/day), in-vivo external ML with different peak dynamic load magnitudes under SN (0N, 6 N, and 12 N), and significantly aged mice with substantially reduced bone density. These results demonstrate the robustness of the proposed method, confirming its reliability in practical applications and its ability to generalize effectively to unseen datasets under varying physiological and experimental conditions. Moreover, the model eliminates intra-operator variability, a limitation commonly observed among human annotators. For comparison, the mean intra-operator ICC values for the manual annotators were 0.99, 0.93, and 0.98, respectively indicating slight inconsistencies even among individual annotators.

We also tested the extraction of the transitional landmarks on the external dataset^12^, on the two groups (risedronate only) and risedronate combined with mechanical loading. The predicted landmarks $Z_{eg}$, $Z_{gp}$ and $Z_{ps}$ were consistently extracted across different bones for both experimental groups, as shown in Fig. 3. For all three landmarks, statistical equivalence was established within a 0.05 mm margin (p $\le$ 0.05) between the manual annotator and the proposed model. Visual assessment confirmed that the extracted cross-sections corresponded accurately to the expected anatomical regions, despite differences in orientation, drug treatments, and microarchitectural morphology (see Fig. 4). Specifically, $Z_{eg}$ was positioned at the transition where a small, non-calcified growth plate cartilage (excluding bridges) begins to disappear, $Z_{gp}$ aligned with the region containing a distinct stripe of non-calcified growth plate cartilage, and $Z_{ps}$ consistently intersected a stripe of primary spongiosa traversing the metaphyseal medulla. These results further validate the model’s reliability in identifying key anatomical transitions across diverse datasets and experimental conditions.

**Table 2:** Three-dimensional morphometric analysis of trabecular bone in (a) the mixed primary–secondary spongiosa from 1 mm distal to the transitional interface between the growth plate and the primary spongiosa ($Z_{gp}$), (b) the secondary spongiosa from 1 mm distal to the transitional interface between the primary and secondary spongiosa ($Z_{ps}$), and (c) the epiphyseal trabecular bone from 0.250 mm proximal to the transitional interface between the epiphyseal bone and the growth plate ($Z_{eg}$) in Dataset 1^22^. Reported morphometric parameters include bone volume fraction (BV/TV, %), trabecular separation (Tb.Sp, µm), and trabecular thickness (Tb.Th, µm), each computed within the corresponding compartment. Values are presented as mean ± standard deviation for all experimental groups. Statistical comparisons were performed using one-way ANOVA followed by Tukey’s HSD post hoc test when the main effect was significant. Superscript letters indicate statistically significant differences from the indicated group: ^a^ PTH0, ^b^ PTH20, ^c^ PTH40, ^d^ PTH80.

Compartment	Group	BV/TV (%)	p	Tb.Th (µm)	p	Tb.Sp (µm)	p
Mixed primary- secondary spongiosa	PTH0	12.98 ± 1.60^b,c,d^	$\le$ 0.001	49.03 ± 1.92 ^d^	$\le$ 0.001	263.5 ± 33.3 ^c,d^	$\le$ 0.001
	PTH20	20.93 ± 0.38^a,c,d^		45.76 ± 2.53 ^d^		233.3 ± 14.2 ^d^	
	PTH40	24.15 ± 1.71^a,b,d^		45.91 ± 1.43 ^d^		214.2 ± 12.6 ^a,d^	
	PTH80	39.30 ± 2.08^a,b,c^		42.33 ± 1.39 ^a,b,c^		140.8 ± 32.7 ^a,b,c^	
Secondary spongiosa	PTH0	8.77 ± 1.12^d^	$\le$ 0.001	49.53 ± 1.76 ^d^	0.008	299.9 ± 35.9	0.287
	PTH20	8.49 ± 0.81^d^		47.08 ± 2.44		306.2 ± 76.2	
	PTH40	9.07 ± 0.83^d^		46.08 ± 2.05		307.6 ± 16.8	
	PTH80	16.57 ± 3.60^a,b,c^		44.40 ± 1.78 ^a^		280.0 ± 35.2	
Epiphyseal bone	PTH0	32.76 ± 2.28^b,c,d^	$\le$ 0.001	71.78 ± 1.52	0.026	246.1 ± 25.6^d^	0.007
	PTH20	39.28 ± 1.69^a,d^		74.15 ± 1.96		227.2 ± 10.5	
	PTH40	39.31 ± 2.51^a,d^		76.00 ± 2.47 ^d^		216.9 ± 24.3	
	PTH80	43.45 ± 2.29^a,b,c^		69.96 ± 4.75 ^c^		187.8 ± 27.3 ^a^	

### Morphometry and statistical analysis

We performed two types of morphological and statistical analyses on the extracted and segmented trabecular compartments of Dataset 1^22^: (i) a 3D analysis based on the entire compartment and (ii) a 2D slice-by-slice cross-sectional analysis.

For the 3D analysis, we reported the morphological parameters bone volume fraction (BV/TV), trabecular thickness (Tb.Th), and trabecular separation (Tb.Sp) in Table 2. The results indicate distinct dose-dependent adaptations across compartments. In the mixed primary–secondary spongiosa (VOI: 1 mm distal from $Z_{gp}$), treatment had a statistically significant effect on BV/TV ($p \le 0.001$). BV/TV increased in a dose-dependent manner across PTH0 (12.98 ± 1.60%), PTH20 (20.93 ± 0.38%), and PTH40 (24.15 ± 1.71%), with a much larger increase at PTH80 (39.30 ± 2.08%). Post hoc analysis showed statistically significant differences between all dose groups, indicating a clear and progressive increase in bone volume fraction within the mixed compartment. Treatment also had a statistically significant effect on Tb.Th ($p \le 0.001$). Tb.Th exhibited a slight, non-significant decrease across PTH0, PTH20, and PTH40, ranging from 49.03 ± 1.92 µm to 45.91 ± 1.43 µm. A further reduction was observed at PTH80 (42.33 ± 1.39 µm), which differed significantly from all other groups, suggesting that trabecular thinning significantly increased at higher PTH doses within this compartment. Tb.Sp was also significantly affected by treatment ($p \le 0.001$). A progressive decrease was observed across doses: from 263.5 ± 33.3 µm at PTH0 to 233.3 ± 14.2 µm at PTH20, 214.2 ± 12.6 µm at PTH40, and reaching 140.8 ± 32.7 µm at PTH80. Post hoc analysis revealed significant differences between PTH0 and both PTH40 and PTH80, as well as between PTH20 and PTH80 and between PTH40 and PTH80, indicating that trabecular separation significantly decreased at higher PTH doses within this compartment.

In the secondary spongiosa (VOI: 1 mm distal from $Z_{ps}$), treatment had a statistically significant effect on BV/TV ($p \le 0.001$). BV/TV remained relatively stable across PTH0, PTH20, and PTH40, ranging from 8.49 ± 0.81% to 9.07 ± 0.83%, with no statistically significant differences between these groups. However, a marked increase was observed at PTH80 (16.57 ± 3.60%, $p \le 0.001$), which differed significantly from all other groups, suggesting that a high PTH dose is required to elicit a robust anabolic response in the defined VOI of the secondary spongiosa. Treatment also had a statistically significant effect on Tb.Th ($p = 0.008$). Tb.Th exhibited a slight, non-significant decrease across PTH0, PTH20, and PTH40 (from 49.58 ± 1.76 µm to 46.08 ± 2.05 µm). A statistically significant reduction was detected at PTH80 compared to the vehicle group PTH0 (44.40 ± 1.78 µm, $p \le 0.001$). In contrast, treatment had no statistically significant main effect on Tb.Sp ($p = 0.287$). Although Tb.Sp increased slightly from PTH0 to PTH40 (299.9 ± 35.9 µm to 307.6 ± 16.8 µm) before decreasing at PTH80 (280.0 ± 35.2 µm), these variations were not statistically meaningful and did not indicate a treatment effect in the secondary spongiosa.

In the epiphyseal bone (VOI: 0.25 mm proximal from $Z_{eg}$), treatment had a statistically significant effect on BV/TV ($p \le 0.001$). BV/TV increased progressively with dose: 32.76 ± 2.28% at PTH0, 39.28 ± 1.69% at PTH20, 39.31 ± 2.51% at PTH40, and 43.45 ± 2.29% at PTH80. Post hoc analysis showed statistically significant differences between PTH0 and all other groups, and between PTH20 and PTH80. Treatment also had a statistically significant effect on Tb.Th ($p = 0.026$). Tb.Th increased from PTH0 to PTH40 (71.78 ± 1.52 µm to 76.00 ± 2.47 µm), followed by a sudden reduction at PTH80 (69.96 ± 4.75 µm). Post hoc analysis revealed significant differences between PTH40 and PTH80 only, suggesting limited structural adaptation of trabecular thickness in the entire compartment. Tb.Sp was also significantly affected by treatment ($p = 0.007$). A gradual decrease was observed across doses: from 246.1 ± 25.6 µm at PTH0 to 227.2 ± 10.5 µm at PTH20, 216.9 ± 24.3 µm at PTH40, and 187.8 ± 27.3 µm at PTH80. Post hoc analysis revealed significant differences between PTH0 and PTH80 only, suggesting a significant compaction of the trabecular network in the epiphyseal compartment at the highest dose.

**Fig. 5:**
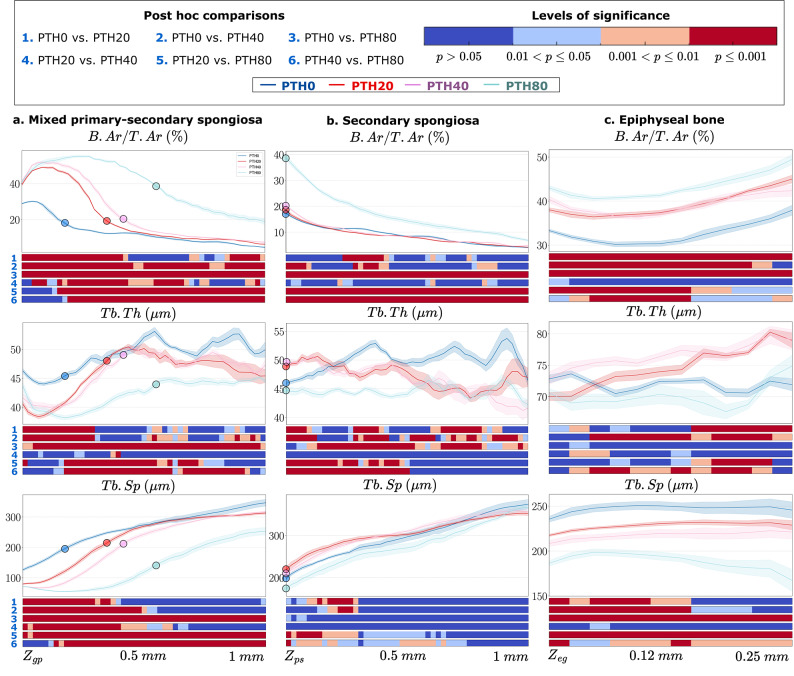
Cross-sectional morphological and statistical analysis of (**a**) the mixed primary-secondary spongiosa from the transitional interface between the growth plate and primary spongiosa ($Z_{gp}$) to 1 mm distally, (**b**) the secondary spongiosa from the transitional interface between the primary and secondary spongiosa ($Z_{ps}$) to 1 mm distally, and (**c**) the epiphyseal trabecular bone from the transitional interface between the epiphyseal bone and the growth plate ($Z_{eg}$) to 0.250 mm proximally in Dataset 1^22^. Reported morphometric parameters include bone area per total area (B.Ar/T.Ar, %), trabecular separation (Tb.Sp, µm), and trabecular thickness (Tb.Th, µm), each computed within the corresponding compartment. The plots display the mean ± standard error, with corresponding heatmaps below indicating post-hoc statistical significance between experimental groups at each depth level: blue $(\hbox {p}> 0.05)$, light blue $(0.01 < \hbox { p} \le 0.05)$, orange $(0.001 < \hbox { p} \le 0.01)$, and red (p $\le$ 0.001). The $Z_{ps}$ landmarks are overlaid in the mixed primary-secondary spongiosa, and at the origin of the secondary spongiosa for each morphological parameter and experimental group to illustrate the axial shift in the transition between primary and secondary spongiosa.

For the 2D analysis, we measured bone area per total area (B.Ar/T.Ar), Tb.Th, and Tb.Sp across all cross-sections within each trabecular compartment, as reported in Fig. 5. In the mixed primary–secondary spongiosa (Fig. 5a), morphometric parameters were evaluated along a 1 mm region distal to $Z_{gp}$. B.Ar/T.Ar exhibited a prominent increase immediately downstream of the growth plate, reaching a peak that both increased in magnitude and shifted distally with increasing PTH dose. This was followed by an inflection point and subsequent decline in all groups. The width and density of the primary spongiosa were dose-dependent, with higher PTH doses inducing an expansion of the high B.Ar/T.Ar region further distally. A significant elevation in the proximal portion of the profile was observed from PTH20 onward compared to the vehicle group. PTH20 and PTH40 displayed similar B.Ar/T.Ar trends along the $z$-axis, while still being statistical different from one another, and converging toward the vehicle profile after approximately 0.5 mm. In contrast, PTH80 maintained a significantly elevated B.Ar/T.Ar across the entire VOI compared to all other groups ($p \le 0.001$, red bars). For Tb.Th, values were lowest in the fine-textured primary spongiosa near $Z_{gp}$, increasing to a local maximum around 0.4–0.6 mm before gradually declining distally. All PTH-treated groups exhibited significantly lower Tb.Th near $Z_{gp}$ compared to the vehicle, reflecting thinner trabeculae in the expanded primary spongiosa. PTH20 and PTH40 followed nearly identical profiles, with minor and localized significant differences relative to PTH0, which diminished with increasing distance from $Z_{gp}$. In contrast, PTH80 induced a pronounced and statistically significant reduction in Tb.Th throughout the VOI relative to all other groups, with the divergence becoming apparent from approximately 0.25 mm distal to $Z_{gp}$. For Tb.Sp, a clear dose-dependent decrease was observed. The most substantial reductions occurred proximally, where all PTH doses differed significantly from the vehicle group. The Tb.Sp profiles for PTH20 and PTH40 had significant local differences proximally, that gradually diminished beyond approximately 0.5 mm from $Z_{gp}$. In contrast, PTH80 exhibited a pronounced and sustained reduction in Tb.Sp across the entire $z$-axis, with highly significant differences from all other experimental groups.

In the secondary spongiosa (Fig. 5b), morphometric parameters were evaluated along a 1 mm region distal to $Z_{ps}$. B.Ar/T.Ar remained largely unchanged across PTH0, PTH20, and PTH40, with minimal local statistical differences. In contrast, PTH80 exhibited a pronounced and consistent increase in B.Ar/T.Ar across the entire VOI compared to all other groups, with strong statistical significance ($p \le 0.001$, red bars). For Tb.Th, PTH20 and PTH40 followed similar profiles along the $z$-axis, exhibiting a statistically significant increase near $Z_{ps}$ compared to the vehicle group PTH0, followed by a progressively significant decrease with depth. No significant differences were observed between PTH20 and PTH40 throughout the VOI. In contrast, PTH80 showed a significant reduction in Tb.Th across the entire VOI compared to PTH0. Tb.Th in PTH80 was also significantly lower than in PTH20 and PTH40 in the proximal region ($p \le 0.001$, red bars), but this difference diminished distally beyond approximately 0.5 mm from $Z_{ps}$, where no statistical significance was detected ($p \ge 0.05$, blue bars). For Tb.Sp, both PTH20 and PTH40 showed a slight increase compared to PTH0 proximally, which was statistically significant up to approximately 0.3 mm from $Z_{ps}$. Beyond this point, Tb.Sp converged with the PTH0 profile showing no statistical different distally ($p \ge 0.05$, blue bars). No significant differences were observed between PTH20 and PTH40 along the $z$-axis. Compared to PTH0, PTH80 exhibited only a marginal decrease in Tb.Sp, with no statistical significance along the $z$-axis. However, Tb.Sp in PTH80 was significantly lower than in PTH20 and PTH40 in the proximal region ($p \le 0.001$, red bars), with profiles converging distally beyond approximately 0.75 mm from $Z_{ps}$ ($p \ge 0.05$, blue bars).

In the epiphyseal trabecular bone (Fig. 5c), morphometric parameters were evaluated along a 0.25 mm region proximal to $Z_{eg}$. B.Ar/T.Ar exhibited a dose-dependent increase along the $z$-axis, with statistically significant increases in all PTH-treated groups compared to the vehicle ($p \le 0.001$, red bars). No significant differences were detected between PTH20 and PTH40 across the axis. In contrast, PTH80 induced a pronounced and sustained increase in B.Ar/T.Ar throughout the VOI, with statistically significant differences from all other PTH doses along the entire $z$-axis. For Tb.Th, PTH20 induced a modest, non-significant increase near $Z_{eg}$, which became more pronounced proximally, reaching statistical significance near the tibial plateau. PTH40 exhibited a similar profile to PTH20, with slightly higher values proximally. Only localized, minor differences were observed between PTH20 and PTH40 along the $z$-axis. In contrast, PTH80 showed slightly lower Tb.Th than the vehicle group throughout the VOI, with almost no local statistically significant differences relative to PTH0. Notably, the highest PTH dose appeared to exert an opposite effect on Tb.Th compared to the lower doses (PTH20 and PTH40), as evidenced by localized statistical significance in the PTH20–PTH80 comparison and near-significant differences between PTH40 and PTH80 along almost the entire VOI.

For Tb.Sp, a clear dose-dependent reduction was observed. The most pronounced differences occurred proximally, where all PTH groups significantly differed from the vehicle, although these differences diminished closer to the tibial plateau. PTH40 showed a slightly lower Tb.Sp than PTH20, but no statistically significant differences were detected between these two groups along the axis. In contrast, PTH80 induced a pronounced and sustained decrease in Tb.Sp throughout the entire VOI, with highly significant differences from all other groups along the entire $z$-axis, suggesting an increase of trabecular number.

Our 2D slice-by-slice cross-sectional and 3D morphological analyses of the trabecular compartments in the mouse tibia enabled robust and efficient comparisons across experimental groups, revealing dose-dependent responses in each compartment and distinct compartment-specific responses.

### Effect of reference level selection on secondary spongiosa morphometry and statistical outcomes

In this section, we examined the impact and limitations of defining the reference level for secondary spongiosa analysis using a fixed offset from the transitional interface between the growth plate and the primary spongiosa ($Z_{gp}$), as traditionally adopted in the literature^2,10,11,22^. We compared this approach with our proposed method, which extracts the transitional interface between the primary and secondary spongiosa ($Z_{ps}$), and uses it as a reference level for VOI definition.

**Fig. 6:**
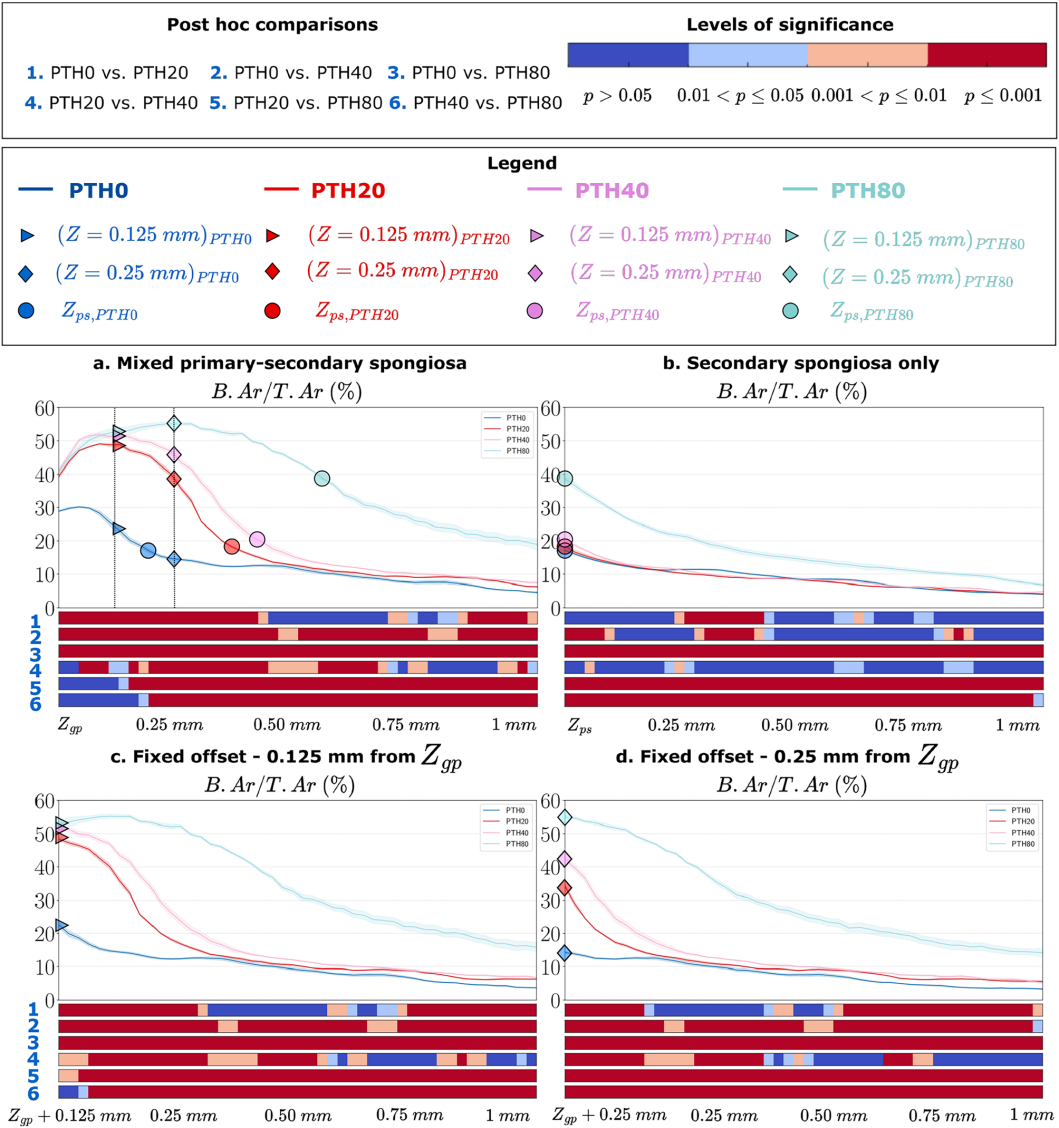
Cross-sectional analysis of the percent bone area per total area (B.Ar/T.Ar) along the $z$-axis in mouse tibiae from Dataset 1^22^, demonstrating the comparison of morphological and statistical interpretations across different volumes of interest. Analyses were performed in the following regions, each spanning 1 mm distally from its respective anatomical reference point: (**a**) the mixed primary-secondary spongiosa from the transitional interface between the growth plate and primary spongiosa ($Z_{gp}$); (**b**) the secondary spongiosa from the transitional interface between the primary and secondary spongiosa ($Z_{ps}$), extracted using our proposed method; (**c**) the metaphyseal trabecular bone starting 0.125 mm from $Z_{gp}$; and (**d**) the metaphyseal trabecular bone starting 0.25 mm from $Z_{gp}$. The plots display the mean ± standard error, with corresponding heatmaps below indicating post-hoc statistical significance between experimental groups at each depth level: blue $(\hbox {p}> 0.05)$, light blue $(0.01 < \hbox { p} \le 0.05)$, orange $(0.001 < \hbox { p} \le 0.01)$, and red (p $\le$ 0.001). Reference levels from fixed offsets of 0.125 mm (triangle) and 0.25 mm (rhombus) from $Z_{gp}$, along with $Z_{ps}$ landmarks (circles), are overlaid in the mixed primary-secondary spongiosa plot (**a**) and their respective corresponding plots.

Specifically, as shown in Fig. 6, we analyzed B.Ar/T.Ar in metaphyseal trabecular bone across four different VOIs, each extending 1 mm distally from their respective reference level: (a) the mixed primary-secondary spongiosa, located directly downstream of $Z_{gp}$; (b) the secondary spongiosa, located downstream of $Z_{ps}$; (c) the trabecular bone, located 0.125 mm downstream of $Z_{gp}$; and (d) the trabecular bone, located 0.25 mm downstream of $Z_{gp}$.

As illustrated in Fig. 6c,d, analyzing the “secondary spongiosa” from a fixed offset distal to the growth plate ($Z_{gp}$) produced markedly different morphological and statistical results compared to analyzing the secondary spongiosa starting from $Z_{ps}$ that we extracted with our proposed method (Fig. 6a). These discrepancies are attributable to the variability in the location of the transitional interface between the primary and secondary spongiosa, which differs across experimental groups and treatment conditions, as previously demonstrated in earlier sections (Figs. 2, 3, 5, 6).

Using a fixed offset to define the reference level can lead to the inclusion of morphologically different trabecular regions. Depending on the treatment group and the chosen offset, the analyzed VOI may fall within: (i) the primary spongiosa (e.g., PTH20, PTH40, PTH80 at 0.125 mm from $Z_{gp}$, and PTH80 at 0.25 mm); (ii) a mixed primary–secondary region (e.g., PTH20 and PTH40 at 0.25 mm); or (iii) the secondary spongiosa alone (e.g., PTH0 at 0.25 mm) (see Fig 6a). This inconsistency introduces substantial variability in the inferred morphometric parameters and statistical interpretations.

A comparison of B.Ar/T.Ar profiles across the different VOIs (Fig. 6b–d) further illustrates this point. When using fixed offsets (c and d), B.Ar/T.Ar exhibits a proximal dose-dependent increase followed by a distal decline. However, this pattern reflects the inclusion of primary or mixed trabecular regions, rather than the mature secondary spongiosa. In contrast, analysis starting from $Z_{ps}$ (Fig. 6b) shows relatively stable B.Ar/T.Ar values across PTH0, PTH20, and PTH40, with little to no significant differences in the proximal region, underscoring the importance of anatomically consistent VOI definition.

This example, derived from a dataset exhibiting pronounced anatomical variability of the transitional interface between the primary and secondary spongiosa, illustrates the methodological limitations of choosing a fixed offset downstream of the growth plate to analyze the secondary spongiosa, and its potential to undermine consistency in the analysis and to produce misleading statistical interpretations. The differences in statistical results are not necessarily incorrect but rather reflect the morphological and statistical outcomes for a fixed region that may include different anatomical regions. It is also important to note that the primary spongiosa is characterized by a fine-textured, thinner, and denser structure compared to the secondary spongiosa^2,11^, and performing statistical tests comparing these two distinct trabecular structures may lack meaningful interpretative value.

Although the transitional interfaces between trabecular compartments are not inherently real, but rather emergent in the context of bone growth, their identification remains necessary for morphometric analysis. The metaphyseal spongiosa represents a dynamic continuum across both spatial and temporal dimensions, with the primary spongiosa positioned at one end of this continuum^2^. Clear region definitions enable meaningful comparisons between animals and treatment groups, ensuring that differences in bone morphology are not confounded by misaligned developmental stages. Consequently, categorizing these regions provides the specificity required for reproducible and consistent morphometric studies.

## Discussion

In this paper, we introduced a deep learning-based framework for consistent analysis of trabecular bone compartments from micro-CT imaging data in the epiphyseal-metaphyseal region of mouse tibiae. This method provides a robust and standardized approach, reducing the inherent inter- and intra-operator variability associated with manual annotation. By ensuring consistent and reproducible results, our proposed method facilitates direct comparisons across studies, promoting standardization in preclinical skeletal research for drug discoveries and bone diseases.

Deep learning is increasingly applied in preclinical research, particularly in high-resolution micro-CT bone imaging. A recent study by Burlutskiy et al.^35^ prepared a high-quality dataset of 3D micro-CT bone scans from 83 mice, focusing on growth plate delineation. The challenge engaged 23 AI teams and demonstrated that diverse computer vision solutions (2D, 2.5D, and 3D) yielded highly similar results. However, while growth plate detection is a crucial step, it is only the first step used to determine the reference point for the extraction of the metaphyseal trabecular compartments and alone is insufficient to fully address the challenges associated with trabecular bone analysis. Our proposed framework extends beyond growth plate detection by classifying all key regions within the epiphyseal-metaphyseal region, including the epiphyseal bone, growth plate, primary spongiosa, and secondary spongiosa, allowing for a direct extraction of the volumes of interest across different studies consistently following current standards^2,10,11^.

We performed region classification using a 2D cross-sectional approach. The decision to adopt a 2D approach was driven by two main considerations: first, this approach aligns well with the extraction of the metaphyseal bone (either secondary spongiosa alone from $Z_{ps}$, or the mixed primary-secondary spongiosa from $Z_{gp}$), which is typically performed in the transverse plane where these transitional interfaces are most clearly defined^2,10,11^. Additionally, as shown in Supplementary Fig. S1, the proposed regional probability distribution method remained robust and consistent across the different PTH groups, effectively capturing the transitional interfaces between compartments within the epiphyseal–metaphyseal region of the mouse tibia, even when the position of these landmarks shifts along the $z$-axis. Second, the 2D cross-sectional method is computationally efficient, a crucial factor when processing high-resolution micro-CT images. Compared to 2.5D and 3D techniques, it reduces the input size, leading to lower computational costs and improved processing speed^35^. Moreover, the same study reported that full 3D convolutions did not improve accuracy over the computationally simpler 2D cross-sectional approach in the delineation of the growth plate^35^.

To develop a robust and generalizable approach, we trained our framework on a diverse dataset encompassing different experimental conditions, including drug treatments and mechanical loading characterized by increased bone mass^22,23^, as well as aged mice with severely reduced trabecular bone^24^. This diversity enabled us to validate the method’s generalizability and robustness in defining standardized VOIs across different experimental studies. We also evaluated the model on an external dataset^12^ that comprised unseen test data from independent studies featuring a different pharmacological treatment (risedronate at 15 µg/kg/day) and mechanical loading conditions, to further assess its generalizability. Despite being trained on a limited dataset of only 40 bone scans, the model consistently demonstrated strong performance across the external dataset, accurately extracting the different compartments within the epiphyseal-metaphyseal region. These results further confirmed the method’s robustness and ability to generalize effectively to new experimental conditions beyond those included in the training data.

The proposed regional distribution probability method assumes that the four compartments are always present in the epiphyseal-metaphyseal region of the tibia. However, this assumption does not always hold. For instance, the growth plate gradually closes with age, although it remains partially open in rodents^36^, and the primary spongiosa may be markedly reduced or even absent in certain cases^24^. In these situations, extracting landmarks such as $Z_{eg}$ and $Z_{gp}$ becomes challenging or even infeasible, as they may not exist. When this occurs, new landmarks can be defined to separate adjacent compartments that remain present (e.g., $Z_{gs}$, the interface between the growth plate and secondary spongiosa), that exists if the primary spongiosa is not present. This limitation does not compromise the model’s overall utility, as the absence of the primary spongiosa is common, and analyses often focus on the secondary spongiosa in such cases^10,12,23,25^. The model demonstrated strong performance in identifying both the secondary spongiosa and the epiphyseal bone, achieving F1-scores of 0.99 and 0.97, respectively, on the test dataset. This ensures that the model remains effective in extracting the two key compartments for further morphological analysis.

We analyzed morphological parameters, both in 3D and in 2D cross-section, of all trabecular compartments within the epiphyseal-metaphyseal region of the mouse tibia. By extracting and segmenting the different trabecular bone compartments, we examined: (i) the mixed primary-secondary spongiosa, from $Z_{gp}$ to 1 mm distally; (ii) the secondary spongiosa alone, starting from $Z_{ps}$ and extending 1 mm distally, representing the mature metaphyseal trabecular bone traditionally analyzed in preclinical bone studies; and (iii) the epiphyseal trabecular bone, extending from $Z_{eg}$ to 0.25 mm proximally. This morphological and statistical analysis revealed distinct dose-dependent responses in the different trabecular compartments, as well as distinct compartment-specific responses for each morphological parameter. Although not directly explored in this paper, 2D cross-sectional profiling can be used to identify local axial regions of significant change in the trabecular bone between experimental groups, guiding subsequent targeted 3D analysis. These approaches are complementary, helping to reveal biologically meaningful spatial patterns in preclinical skeletal research.

This analysis highlighted a methodological limitation in conventional morphometric studies of the secondary spongiosa: the use of fixed-offset reference levels from the growth plate ($Z_{gp}$) to define the start of the VOI for analysis. Our results demonstrate that the transition between the primary and secondary spongiosa varies across animals and treatment conditions, particularly under strong anabolic stimuli such as PTH. In Dataset 1^22^, for example, PTH treatment markedly extended the primary spongiosa, shifting the offset of the secondary spongiosa distally. Applying a fixed offset downstream of the growth plate in such cases resulted in inclusion of the primary or mixed primary-secondary regions, thereby altering the morphological and statistical outcomes, as illustrated in Fig 6. This anatomical variability is less pronounced but still present in experimental groups that do not involve strong anabolic effects, such as those subjected to mechanical loading under moderate peak forces^23^ or receiving antiresorptive treatment (e.g., risedronate)^12^. Even in these cases, fixed-offset reference levels may introduce subtle misalignments across animals. In contrast, automated detection of the transitional interface between the primary and secondary spongiosa ($Z_{ps}$) and its use as a reference level enables consistent analysis of the secondary spongiosa across different experimental groups and datasets. This approach improves reproducibility and ensures that group-level comparisons are performed on structurally equivalent trabecular bone compartments.

Although the datasets analyzed in this study varied in biological and experimental conditions, all imaging was performed using the same microCT system. Consequently, further work is required to evaluate the method’s performance across scanners with differing hardware specifications and resolutions. This limitation could potentially be addressed through transfer learning strategies, which have demonstrated efficacy in adapting models across imaging domains with limited labeled data^37,38^. It is also important to note that mouse and rat bones fall well within the imaging capabilities of all commercially available microCT systems in terms of resolution (5–10 µm voxel size^39^) and X-ray energy (0.5–1 mm Al filter, 40–80 kV). Therefore, substantial differences in image characteristics (e.g., signal-to-noise ratio, resolution) are not typically expected^40,41^. Such differences as evident in the literature often arise from non-optimal use of a particular scanner rather than differences between scanners. To minimize such variability, researchers are encouraged to; optimize their scan protocols, regularly system their microCT scanner, undertake regular calibration, conduct flat-field corrections for each individual sample, determine optimal angular sampling and X-ray signal strength, select appropriate filters and voltage, and apply corrections for common artifacts such as ring artifacts and beam hardening^9,42^.

Further improvements could be achieved by expanding the training dataset, as deep learning models benefit from greater data diversity and volume. Incorporating additional experimental conditions, such as younger mice, genetically modified strains (e.g., Sost knockout), and wild-type mice with varying genetic backgrounds, along with different micro-CT resolutions and orientations, would enhance the model’s ability to generalize across a broader range of biological and imaging variations. Moreover, this framework could be adapted to other rodent models or extended to the femur, as the compartment landmarks share similar characteristics^11^. Additionally, more sophisticated frameworks that combine 2D and 2.5D approaches could potentially achieve a better balance between performance and efficiency, as suggested by recent findings^35^.

Our results demonstrated the capability of the proposed approach to automatically detect and extract the different trabecular bone compartments within the epiphyseal-metaphyseal region of the mouse tibia, enabling comprehensive and consistent morphological and statistical analysis. Standardized trabecular compartment extraction and segmentation enables consistent comparisons between studies, enhancing the reliability of trabecular bone adaptation analyses under different experimental conditions, such as pharmacological interventions and mechanical loading, and ensures accurate morphological and statistical assessment.

In conclusion, our deep-learning framework provides a significant advancement in automated trabecular bone analysis in murine models, addressing the limitations of manual annotation and inconsistent VOI definition. Manual processing of high-resolution micro-CT scans is highly labor-intensive and time-consuming, particularly due to the large image volumes and the substantial computational resources required. The integration of deep learning in preclinical bone research has the potential to accelerate the pace of advances in this field, improving reproducibility and accelerating discoveries related to bone remodeling and treatment responses. By ensuring a standardized and automated workflow, our method paves the way for more reliable and interpretable morphometric analyses, ultimately contributing to advancements in degenerative bone diseases and drug discoveries.

## Methods

### Defining the VOIs of the trabecular compartments

The proximal tibia can be divided into four distinct compartments progressing from proximal to distal when viewed from the transverse plane. The first region is the epiphyseal bone, which lies directly beneath the articular cartilage. This region is dense and provides critical structural support to the overlying cartilage, facilitating load distribution across the joint^43^. Below the epiphysis is the growth plate, a tortuous and chondrocyte-rich cartilaginous structure that serves as the primary site for longitudinal bone growth during development. The distal edge of the growth plate comprises calcified cartilage in the process of being replaced by bone, marking the transition to the primary spongiosa.

**Fig. 7:**
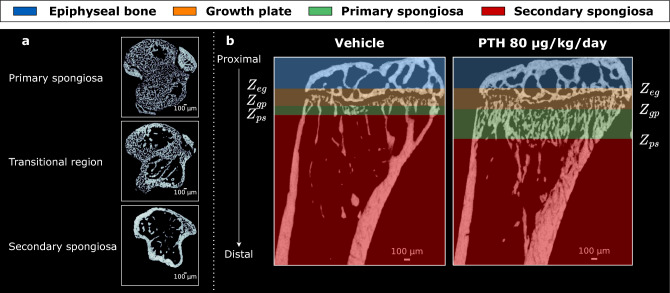
Defining the volumes of interest of the trabecular compartments in the epiphyseal-metaphyseal region of the mouse tibia. (**a**) Micro-CT visualization (5 µm) of cross-sections along the proximal tibial metaphysis of a 17-week-old female C57BL/6 mouse, illustrating the primary spongiosa, the transitional region between primary and secondary spongiosa, and the secondary spongiosa. The animal received a low dose of intermittent parathyroid hormone (PTH; 20 µg/kg/day for 4 weeks; Dataset 1^22^). (**b**) Coronal micro-CT view of the proximal tibia from a vehicle-treated mouse (left) and a mouse treated with a high dose of intermittent PTH (80 µg/kg/day for 4 weeks; Dataset 1^22^) (right), showing the different compartments: epiphyseal bone, growth plate, primary spongiosa, and secondary spongiosa. $Z_{eg}$ represents the transitional interface between the epiphyseal bone and the growth plate. $Z_{gp}$ represents the transitional interface between the growth plate and the primary spongiosa, and $Z_{ps}$ represents the transitional interface between the primary and secondary spongiosa.

The primary spongiosa consists of immature, finely structured trabeculae that occupy the bone medulla^11^. These trabeculae are a mix of bone and residual cartilage, contributing to early bone formation directly beneath the growth plate. Moving distally, the primary spongiosa gradually disappears, giving way to the secondary spongiosa, the final region in this compartment (see Fig. 7a). The secondary spongiosa consists of mature trabecular bone, where the cartilaginous matrix has been fully replaced by bone tissue. The transitional interface between the primary and secondary spongiosa $Z_{ps}$ has traditionally been considered a reference level in trabecular bone analysis^10,11^, although this view has been challenged more recently^2^.

Focusing on the secondary spongiosa is primarily justified by two key biological considerations. First, the remodeling kinetics in this region occur on a larger spatiotemporal scale and involve basic multicellular units, which resemble the remodeling processes in adult human bone. This similarity lends translational relevance to analyses in murine models. Second, the secondary spongiosa persists for a longer duration of the experiment compared to the newly formed primary spongiosa, making it a more stable and representative region for evaluating long-term bone adaptation. Beyond these biological justifications, analyzing the secondary spongiosa is also practical and convenient. This region is easier to differentiate from the cortical bone, thereby enabling simplified analysis. However, defining the offset distance can become complex in studies involving preclinical animal models of different sizes, where rescaling may be required to account for size and growth differences. The distinction between the primary and secondary spongiosa was recently questioned by Salmon et al.^2^, who investigated the validity of including mixed primary–secondary spongiosa trabecular bone, extending the analyzed volume to start from $Z_{gp}$ instead of $Z_{ps}$. In this study, we distinguished between these two compartments to enable analyses of either the secondary and primary spongiosa separately or as a mixed primary–secondary spongiosa, depending on the research question.

To ensure consistent and accurate analysis, the growth plate serves as a reference level for defining the VOI in metaphyseal trabecular bone studies. In young and mature rodents, the growth plate is clearly visible as a cartilage seam. The reference level, denoted as $Z_{gp}$, corresponds to the transitional interface between the growth plate and the primary spongiosa, and serves as the starting point for defining the VOI. From this reference, the “offset” and “height” of the VOI are determined. The offset corresponds to the distance translated along the bone’s longitudinal axis to reach the secondary spongiosa at $Z_{ps}$ where mature trabecular bone dominates. Determining the appropriate offset and VOI dimensions is influenced by various factors, including age, strain, sex, drug treatments, mechanical loading, and/or surgical interventions in the animals’ studies. Fig. 7b shows a coronal view of the epiphyseal-metaphyseal region of the proximal tibia with the different landmarks $Z_{eg}$, $Z_{gp}$, and $Z_{ps}$. As illustrated in Fig. 7b, in the case of high-dose PTH treatment (80 µg/kg/day), these landmarks can exhibit significant axial shifts, which underscores the limitations of using a “fixed offset” and further motivates the need for automated detection of these transitional landmarks.

This study therefore employed the use of consistently reproducible anatomical landmarks to extract all the trabecular compartments with the epiphyseal-metaphyseal region of the mouse tibia. Annotations were performed as follows: three independent investigators were shown reconstructed micro-CT scans of mouse tibiae in transverse plane, starting at the mid-diaphysis and moving proximally slice by slice. The scan orientation was different between samples to ensure better training of the deep-learning model, and speed of proximal movement was investigator-controlled. Investigators identified, in order: 1) the region depicting the transition between secondary and primary spongiosa ($Z_{ps}$). This was defined as the distal-most transverse slice in which a stripe of primary spongiosa was seen to traverse the metaphyseal medulla, uninterrupted from anterior to posterior (see Fig. 7.a). 2) The region depicting the transition between the primary spongiosa and growth plate ($Z_{gp}$). This was defined as the distal-most slice in which a stripe of non-calcified (excluding bridges) growth plate cartilage^44^ was seen to traverse the metaphyseal medulla, uninterrupted from anterior to posterior. $Z_{ps}$ and $Z_{gp}$ were defined as reference levels for the secondary spongiosa and the growth plate, respectively, as traditionally adopted in the literature^10,11^. These anatomical landmarks represent recurring morphological signatures in rodents, including rats and mice^11^. 3) The region depicting the transition from growth plate to epiphysis ($Z_{eg}$). This was defined as the proximal-most slice in which any non-calcified (excluding bridges) growth plate cartilage was still visible. In contrast to $Z_{ps}$ and $Z_{gp}$, which are well established in histological and imaging studies, the choice of $Z_{eg}$ reflects a novel yet biologically grounded criterion. The tibial epiphysis is demarcated distally by the growth plate, whose structural integrity and fusion status are key endpoints in studies of longitudinal growth and skeletal maturation^45^. By defining $Z_{eg}$ as the most proximal (i.e., superior) slice where any growth plate cartilage is still visible, we ensure a clear anatomical transition: all slices distal to this point definitively contain growth plate tissue, while all proximal slices do not. This minimizes risk of VOI misclassification and guarantees consistent compartmental labeling. Moreover, this definition enhances reproducibility. The identification of the first slice with definitive cartilage presence is more objective and less susceptible to inter-operator variability. This approach is supported by recent work in anatomical imaging, which found that “most superior visible point” landmarks yield higher inter-rater reliability than less well-defined alternatives^46^. Therefore, the adoption of $Z_{eg}$ balances anatomical relevance with methodological robustness, contributing to the consistency of our automated pipeline.

Annotation was repeated by all investigators, with scans shown in a different sequence on repetition. These landmarks were identifiable in all groups regardless of orientation, treatment, and age differences, confirming that they are suitable for use in a diverse array of murine tibial bones.

### Datasets

We trained and validated the model using three high-resolution micro-CT datasets from studies employing the C57BL/6 mouse model, each featuring distinct experimental conditions. This strain is widely used for investigating bone adaptation to mechanical loading and pharmacological treatments.

**Dataset 1**^22^: This dataset includes virgin, female C57BL/6 mice aged 13 weeks at the start of the experiment. Mice received daily subcutaneous injections of intermittent parathyroid hormone (PTH) over a 4-week period, with four treatment groups based on dosage (n=5 per group): vehicle control (0 µg/kg/day), low (20 µg/kg/day), medium (40 µg/kg/day), and high (80 µg/kg/day). At 17 weeks of age, mice were euthanized, and their tibiae and ulnae were harvested. High-resolution micro-CT imaging was performed with a Bruker Skyscan 1172 desktop micro-CT with the following configuration: voxel size 5 µm, source voltage 40 kV, source current 230 µA, 0.5 mm aluminium filter, rotation step 0.600 $\phantom{0}^\circ$ and image format bmp.

**Dataset 2**^23^: This dataset includes female C57BL/6 mice aged 17 weeks. Sciatic neurectomy (SN) was performed on the right leg to induce disuse. From day 5 to day 19 post-surgery, the right tibia was subjected to external mechanical loading. Three groups were analyzed based on loading magnitude (n=5 per group): 0 N (SN only), 6 N, and 12 N. Only the right tibia was analyzed. High-resolution micro-CT imaging was performed with a Bruker Skyscan 1172 desktop micro-CT with the following configuration: voxel size 5 µm, source voltage 49 kV, source current 200 µA, 0.5 mm aluminium filter, rotation step 0.600 $\phantom{0}^\circ$ and image format bmp.

**Dataset 3**^24^: This dataset includes aged (19-month-old) female C57BL/6 mice (n=5) used to evaluate age-related bone loss. High-resolution micro-CT imaging was performed with a Bruker Skyscan 1172 desktop micro-CT with the following configuration: voxel size 4.78 µm, source voltage 49 kV, source current 200 µA, 0.5 mm aluminium filter, rotation step 0.600 $\phantom{0}^\circ$ and image format bmp.

**External dataset**^12^: After evaluating generalization performance, we retrained the final model on all three internal datasets and tested it on this external dataset. This dataset consists of 17-week-old female C57BL/6 mice treated with daily subcutaneous injections of risedronate at a dose of 15 µg/kg/day. Two groups were included (n=5 per group): one group received risedronate alone, while the other received risedronate in combination with mechanical loading. High-resolution micro-CT imaging was performed with a Bruker Skyscan 1172 desktop micro-CT with the following configuration: voxel size 5 µm, source voltage 40 kV, source current 250 µA, 0.5 mm aluminium filter, rotation step 0.500 $\phantom{0}^\circ$ and image format bmp.

All mice were obtained from Charles River Laboratories, Inc. (Margate, UK). Mice were euthanized using isoflurane. All procedures complied with the UK Animals (Scientific Procedures) Act 1986 and were reviewed and approved by the ethics committee of the Royal Veterinary College (London, UK). This study is reported in accordance with the ARRIVE guidelines. Additional details regarding the animals, the experimental designs, and ethical approvals have been previously reported in the original studies^12,22–24^.

### Deep learning-based region classification

**Fig. 8:**
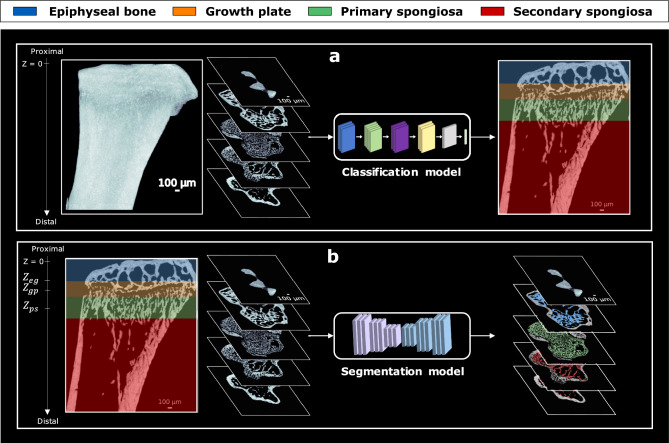
Proposed deep learning-based framework for the automated analysis of trabecular bone compartments in the epiphyseal-metaphyseal region of the mouse tibia. (**a**) Deep learning-based classification of the epiphyseal-metaphyseal region into four compartments: epiphyseal bone, growth plate, primary spongiosa, and secondary spongiosa, for standardized VOI extraction. (**b**) Deep learning-based segmentation of the epiphyseal trabecular bone, primary spongiosa, and secondary spongiosa, using the consistent VOIs extracted in (**a**).

We developed a deep learning-based framework to classify the epiphyseal-metaphyseal region of the mouse tibia into four distinct regions for the analysis of trabecular bone, as illustrated in Fig. 8a. This approach framed the problem as a 2D image classification task, where the 3D micro-CT images of the proximal tibia are split into cross-sectional slices along the $z$-axis. These slices are analyzed and classified into four region classes: epiphyseal bone, growth plate, primary spongiosa, and secondary spongiosa. We employed the ResNet architecture as the backbone of the classification model^47^, a widely used convolutional neural network known for its robust feature extraction capabilities. Pretrained weights from the ImageNet dataset were used to leverage transfer learning by enabling the model to start with features already learned on a large, diverse dataset, which reduces training time and improves model accuracy. The dataset underwent the following preprocessing steps to ensure consistency and efficiency: tibiae were segmented from the sample-holder assembly using Otsu’s thresholding^48^; isolated artifacts were removed by retaining only the largest connected component; images were cropped to the tibia bounding box, normalized to [0,1] intensity range, and resized to 384 $\times$ 384 pixels using linear interpolation. We trained the model using a cross-entropy loss function, optimized with stochastic gradient descent (SGD), with a learning rate of 10-3 and a batch size of 64. The aggregation of the multiple annotations was performed using majority voting^49^. The dataset comprised 28,155 2D micro-CT slices. To evaluate generalization performance, we employed 5-fold cross-validation^50^. At each iteration, the model was trained on four folds and validated on the remaining one. The classification performance was then assessed on the held-out fold, and results were averaged across all five folds to obtain a robust estimate of generalization performance. Model evaluation was conducted using precision, recall, and the F1-score, alongside confusion matrices, to assess classification performance. Precision is the proportion of predicted positives that are actually positive, recall is the proportion of all actual positives that were classified correctly, and F1-score is the harmonic mean of precision and recall.

### Regional probability distribution

**Fig. 9:**
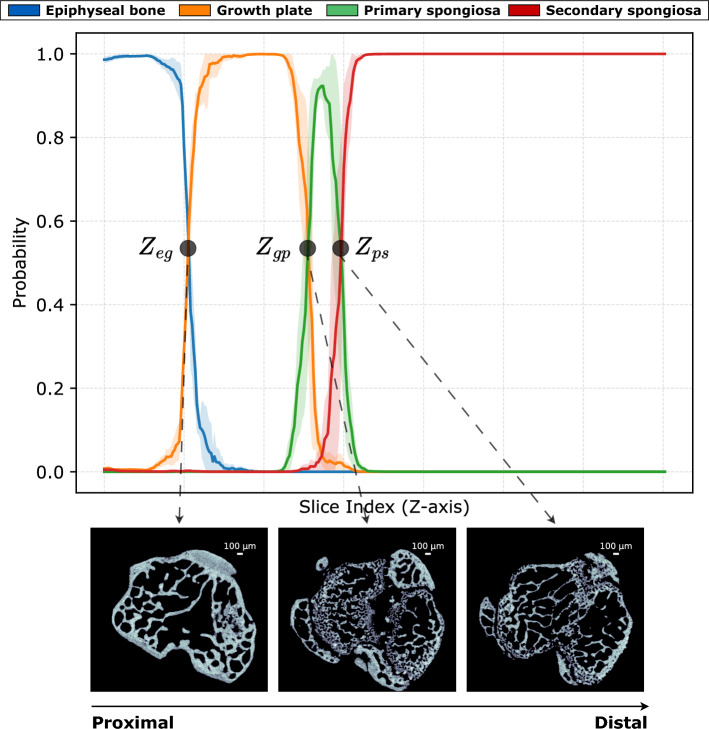
The regional probability distribution method for extracting the different trabecular compartments within the epiphyseal-metaphyseal region of the mouse tibia. A deep learning model classifies 2D micro-CT cross-sections along the $z$-axis into four compartments: epiphyseal bone, growth plate, primary spongiosa, and secondary spongiosa. The model processes the 3D image slice by slice, and extracts class probability profiles to identify the three transitional landmarks: $Z_{eg}$, the transitional interface between the epiphyseal bone and the growth plate; $Z_{gp}$, the transitional interface between the growth plate and the primary spongiosa; and $Z_{ps}$, the transitional interface between the primary spongiosa and the secondary spongiosa. The example shown illustrates the mean ± standard deviation of the predicted class probabilities in the vehicle-treated (PTH0) group from Dataset 1^22^.

We applied our trained deep learning model to extract the different trabecular compartments within the epiphyseal-metaphyseal region of the mouse tibia. To achieve this, we proposed a regional probability distribution method, illustrated in Fig. 9.

First, the model’s output logits are passed through a softmax function to obtain the class probabilities for each slice. We applied a rolling median filtering (window = 0.05 mm) to the raw probabilities to reduce noise and ensure slice connectivity, generating smooth probability distributions along the $z$-axis, from the proximal to the distal region.

Compartment classification focused on epiphyseal bone ($C = 0$), growth plate ($C = 1$), primary spongiosa ($C = 2$), and secondary spongiosa ($C = 3$). Starting from the proximal region, the model shows a high probability for detecting the epiphyseal bone ($C = 0$) in the epiphyseal region, while the probabilities for other classes remain close to zero. As we move distally toward the growth plate ($C = 1$), the probability of epiphyseal bone detection begins to decrease, while the probability for the growth plate increases. The transitional interface between the epiphyseal bone and the growth plate is defined by the landmark $Z_{eg}$, as specified in Eq. 1:

1 $$\begin{aligned} Z_{eg} = \min (z) \quad \text {where} \quad P(z, C=0) = P(z, C=1) \end{aligned}$$

As we continue distally, the probability of the growth plate reaches its peak and then begins to drop as we transition into the primary spongiosa ($C = 2$). The transitional interface between the growth plate and the primary spongiosa is defined by the landmark $Z_{gp}$, as specified in Eq. 2:

2 $$\begin{aligned} Z_{gp} = \min (z) \quad \text {where} \quad P(z, C=1) = P(z, C=2) \end{aligned}$$

Further along the distal axis, as we get closer to the secondary spongiosa ($C = 3$), the probability of the primary spongiosa declines while the probability of the secondary spongiosa increases. The transitional interface between the primary spongiosa and the secondary spongiosa is defined by the landmark $Z_{ps}$, as specified in Eq. 3:

3 $$\begin{aligned} Z_{ps} = \min (z) \quad \text {where} \quad P(z, C=2) = P(z, C=3) \end{aligned}$$

Beyond $Z_{ps}$, the model confidently predicts secondary spongiosa, with probabilities approaching 1 as we progress distally. This probability-based approach allows precise classification of the epiphyseal-metaphyseal region in the mouse tibia, facilitating the analysis of bone morphology and structure. In practice, $Z_{eg}$ is measured from proximal to distal, $Z_{ps}$ from distal to proximal, and $Z_{gp}$ within the region bounded by $Z_{ps}$ and $Z_{eg}$.

The landmark $Z_{eg}$ marks the starting point for trabecular bone analysis in the epiphyseal compartment. In the metaphyseal compartment, $Z_{gp}$ defines the starting point for analyzing mixed primary–secondary spongiosa, while $Z_{ps}$ is used when analyzing the secondary spongiosa alone.

To evaluate the accuracy of the model-predicted transitional landmarks ($Z_{eg}$, $Z_{gp}$, and $Z_{ps}$), we compared the model outputs with manual annotations performed by three annotators. These annotators independently identified each landmark in all samples of each dataset. For each landmark and experimental group, we assessed the agreement between pairs of human annotators, and between the model and each annotator, using the Two One-Sided Tests (TOST)^51^ for equivalence testing. We applied a Bonferroni correction to control for multiple comparisons. Equivalence was concluded when the adjusted p-value was $\le 0.05$. The model was considered statistically equivalent to human annotations if its predictions were within the defined margin relative to at least two of the three annotators.

### Morphometry and statistical analysis

To ensure consistent anatomical orientation across all samples, each tibia was spatially aligned by computing its principal axis via principal component analysis (PCA) of the segmented bone volume. The first principal component, representing the direction of greatest variance along the tibial shaft, was then aligned to the global $z$-axis using a rigid transformation. To constrain rotation in the $x-y$ plane, the tibial ridge was detected, within the proximal 65–75% segment of the tibial length, by computing the furthest point from the centroid in each slice of the segment. Each sample was rotated such that the mean ridge orientation aligned with the global $x$-axis. This registration procedure standardized the orientation of all samples, enabling consistent cross-sectional analysis across samples.

After consistently extracting the volumes of interest for the metaphyseal and epiphyseal bone across all experimental groups, we segmented the trabecular bone in both compartments using DBAHNet, a hybrid deep learning network designed for the segmentation of high-resolution 3D micro-CT bone scans^33,34^. Specifically, DBAHNet was applied to the epiphyseal-metaphyseal region of the mouse tibia to segment the secondary spongiosa, the primary spongiosa, and the epiphyseal trabecular bone (see Fig. 8b). After segmenting the trabecular bone, we measured standardized morphological parameters^9^ for all the trabecular compartments in the mouse tibia. Specifically, we analyzed morphological parameters, both in 3D and in 2D cross-section, in the secondary spongiosa from $Z_{ps}$ to 1 mm distally, the mixed primary-secondary spongiosa from $Z_{gp}$ to 1 mm distally, and the epiphyseal trabecular bone from $Z_{eg}$ to 0.25 mm proximally.

We performed a 3D morphological analysis on the entire compartments, by measuring the BV/TV, Tb.Th, and Tb.Sp. We applied a one-way ANOVA to assess main treatment effects. If the omnibus test was significant, we performed post hoc pairwise comparisons using Tukey’s HSD^52^,, controlling the family-wise error rate (FWER) at 5%. Statistical assumptions were evaluated prior to hypothesis testing. The normality of residuals was assessed using the Shapiro–Wilk test^53^, and the homogeneity of variance across groups was tested using Levene’s test^54^.

Complementing the 3D morphological analysis, we performed a 2D cross-sectional morphological analysis, by measuring B.Ar/T.Ar, Tb.Th, and Tb.Sp. We computed both Tb.Th and Tb.Sp using a 3D fast local thickness algorithm^55^. At each depth level, we applied a one-way ANOVA to assess main treatment effects. If the omnibus test was significant, we performed post hoc pairwise comparisons using Games-Howell post-hoc comparisons^56^. Specifically, the morphological parameters were binned along the $z$-axis at fixed depth levels (I = 20 µm), and statistical significance was assessed at each $z$-axis depth level for each trabecular compartment separately to compare differences between experimental groups along the entire length of the compartments. The 20 µm step size was empirically chosen to provide higher spatial sampling than the original 5 µm resolution, while minimizing sensitivity to potential misalignments. The cross-sectional statistical analysis is reported through statistical bars indicating significance levels between the different groups, as illustrated in Fig. 5. To enhance interpretability, a color-coded scheme is employed: blue for $\hbox {p}> 0.05$ (not significant), light blue for $0.01 < \hbox { p} \le 0.05$, light orange for $0.001 < \hbox { p} \le 0.01$, and red for p $\le$ 0.001.

This representation allows for a complete comparison of morphometric variations across the different experimental groups, revealing local spatially resolved and volumetric differences within the different trabecular architectures of the mouse tibia.

## Supplementary Information

Supplementary Information.

## Data Availability

All data needed to evaluate the conclusions in the paper are present in the paper. The codes are available at: https://github.com/bigfahma/Trab_analysis_microct. The Micro-CT datasets used are available from P.P. upon reasonable request.
