# Supplementary material for: A deep learning-based framework for standardized analysis of trabecular bone compartments from micro-CT imaging data in the mouse tibia
Source: Sci Rep. 2025 Oct 14;15:35807. doi: 10.1038/s41598-025-19698-w (PMC12521361; doi:10.1038/s41598-025-19698-w)
Supplement: Supplementary file 1 — Supplementary Information. [file 41598_2025_19698_MOESM1_ESM.pdf]

Supplementary Information for:
A deep learning-based framework for standardized analysis of trabecular bone compartments from micro-CT imaging data in the mouse tibia

Amine Lagzouli[1,2,*], Lucinda Evans[3], Mark Hopkinson[3], Aikta Sharma[3,4], Natalia M. Castoldi[1], Davide Fontanarosa[7,8], Maria Antico[9], David M.L. Cooper[5], Alice Othmani[6], Vittorio Sansalone[2], Phil Salmon[10], Andrew A. Pitsillides[3], and Peter Pivonka[1,*]

[1]School of Mechanical, Medical, and Process Engineering, Queensland University of Technology, Gardens Point Campus, 2 George St, Brisbane, QLD 4000, Brisbane, Australia
[2]Univ Paris Est Creteil, Univ Gustave Eiffel, CNRS, UMR 8208, MSME, F-94010 Créteil, France
[3]Department of Comparative Biomedical Sciences, Royal Veterinary College, London, NT1 0TU, UK
[4]Department of Mechanical Engineering, University College London, UK
[5]Department of Anatomy, Physiology, and Pharmacology, University of Saskatchewan, Saskatoon, Saskatchewan, Canada
[6]Université Paris-Est Créteil (UPEC), LISSI, Vitry sur Seine 94400, France
[7]School of Clinical Sciences, Queensland University of Technology, Gardens Point Campus, 2 George St, Brisbane, QLD 4000, Australia
[8]Centre for Biomedical Technologies (CBT), Queensland University of Technology, Brisbane, QLD 4000, Australia
[9]CSIRO Health and Biosecurity, The Australian eHealth Research Centre, Herston, QLD 4029, Australia
[10]Bruker Belgium (microCT), Preclinical Imaging, Kontich, Belgium
[*]Corresponding authors. Email: aminelagzouli02@gmail.com (A.L.); peter.pivonka@qut.edu.au (P.P.)

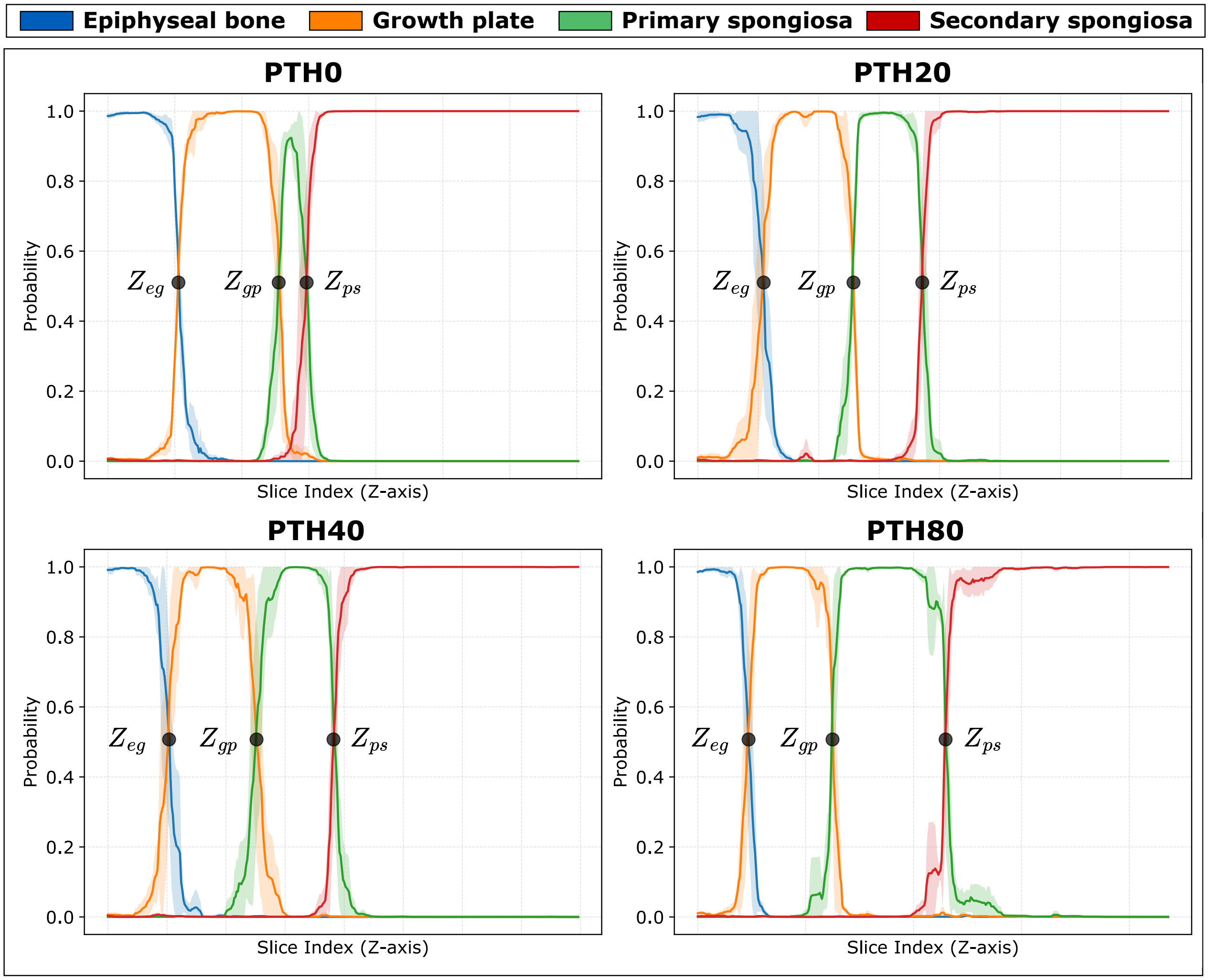

Supplementary Figure S1. The regional probability distribution method for extracting the different trabecular compartments within the epiphyseal-metaphyseal region of the mouse tibia. A deep learning model classifies 2D micro-CT cross-sections along the z-axis into four compartments: epiphyseal bone, growth plate, primary spongiosa, and secondary spongiosa. The model processes the 3D image slice by slice, and extracts class probability profiles to identify the three transitional landmarks: Z_{eg}, the transitional interface between the epiphyseal bone and the growth plate; Z_{gp}, the transitional interface between the growth plate and the primary spongiosa; and Z_{ps}, the transitional interface between the primary spongiosa and the secondary spongiosa. This figure illustrates the mean ± standard deviation of the predicted class probabilities across all four treatment groups (PTH0, PTH20, PTH40, and PTH80 µg/kg/day) from Dataset 1[1], demonstrating the consistency and robustness of the proposed method in detecting the transitional landmarks across different experimental groups.

References

1. Sugiyama, T. *et al.* Mechanical loading enhances the anabolic effects of intermittent parathyroid hormone (1–34) on trabecular and cortical bone in mice. *Bone* **43**, 238–248 (2008).
